# Supplementary material for: E. coli Biomolecules Increase Glycolysis and Invasive Potential in Lung Adenocarcinoma
Source: Cancers (Basel). 2025 Jan 24;17(3):380. doi: 10.3390/cancers17030380 (PMC11815989; doi:10.3390/cancers17030380)
Supplement: Supplementary file 1 [file cancers-17-00380-s001.zip › cancers-3350731-supplementary.pdf]

Figure 2B

Original Uncropped blots

Manuscript Figure

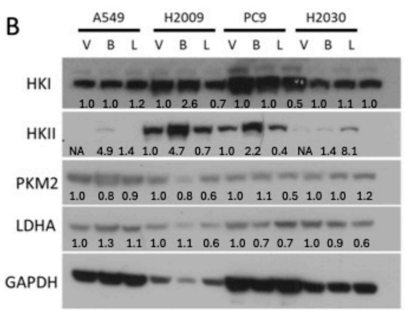

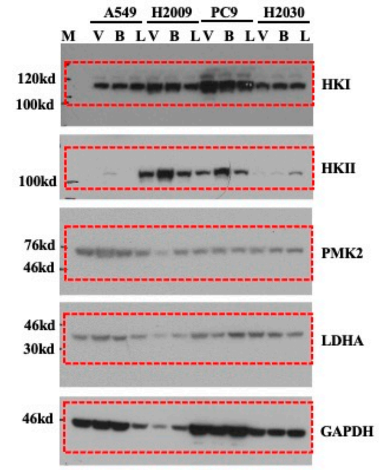

Note: M is Molecular weight marker

Figure 4B

Original Uncropped blots

Manuscript Figure

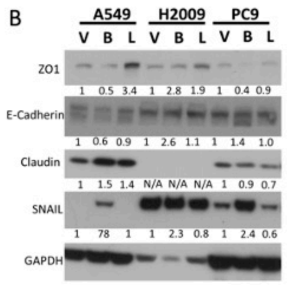

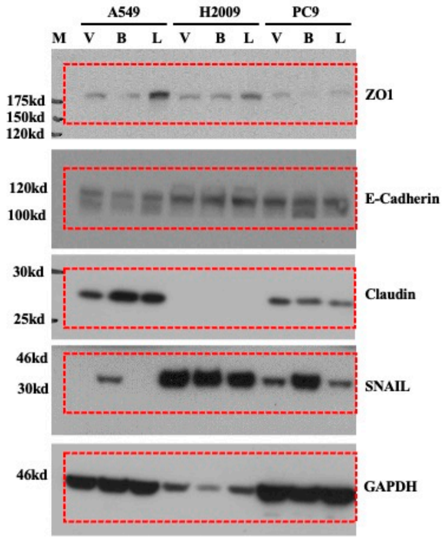

Note: M is Molecular weight marker

Figure 5B and 5C

Original Uncropped blots

Manuscript Figure

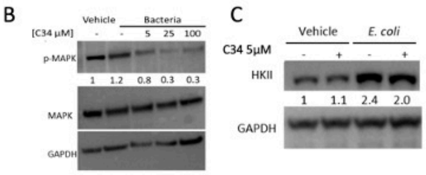

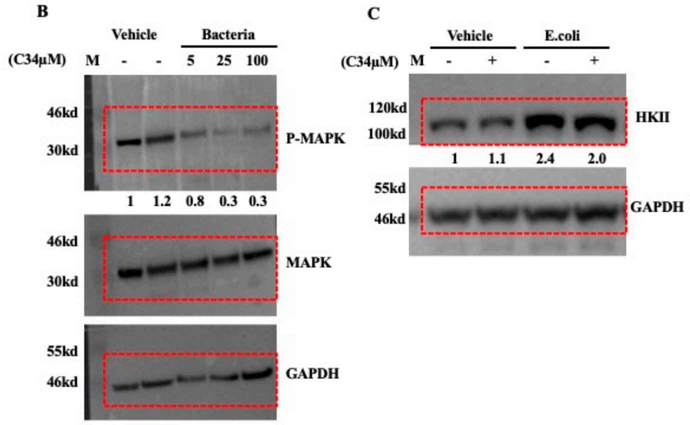

Note: M is Molecular weight marker

Figure 6A

Original Uncropped blots

Manuscript Figure

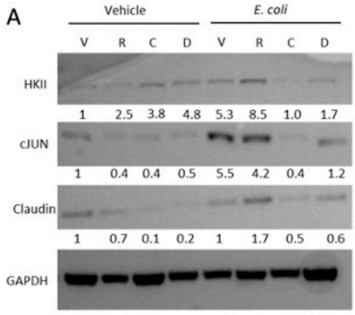

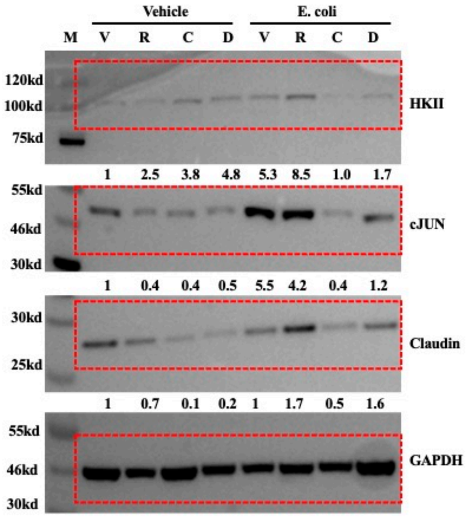

Note: M is Molecular weight marker
